# The efficacy and safety of high-dose isoniazid-containing therapy for multidrug-resistant tuberculosis: a systematic review and meta-analysis

**DOI:** 10.3389/fphar.2023.1331371

**Published:** 2024-01-08

**Authors:** Ming Zhou, Ai-Mei Liu, Xiao-Bing Yang, Cui-Ping Guan, Yan-An Zhang, Mao-Shui Wang, Ya-Li Chen

**Affiliations:** ^1^ Department of Laboratory Medicine, Chest Hospital of Guangxi Zhuang Autonomous Region, Liuzhou, Guangxi, China; ^2^ Department of Infectious Diseases, Chest Hospital of Guangxi Zhuang Autonomous Region, Liuzhou, Guangxi, China; ^3^ Department of Lab Medicine, Shandong Public Health Clinical Center, Shandong University, Jinan, Shandong, China; ^4^ Shandong Key Laboratory of Infectious Respiratory Disease, Jinan, Shandong, China; ^5^ Department of Cardiovascular Surgery, Shandong Public Health Clinical Center, Shandong University, Jinan, Shandong, China

**Keywords:** high dose, isoniazid, treatment success, death, MDR-TB

## Abstract

**Objectives:** Accumulating evidence are available on the efficacy of high-dose isoniazid (INH) for multidrug-resistant tuberculosis (MDR-TB) treatment. We aimed to perform a systematic review and meta-analysis to compare clinical efficacy and safety outcomes of high-dose INH- containing therapy against other regimes.

**Methods:** We searched the following databases PubMed, Embase, Scopus, Web of Science, CINAHL, the Cochrane Library, and ClinicalTrials.gov. We considered and included any studies comparing treatment success, treatment unsuccess, or adverse events in patients with MDR-TB treated with high-dose INH (>300 mg/day or >5 mg/kg/day).

**Results:** Of a total of 3,749 citations screened, 19 studies were included, accounting for 5,103 subjects, the risk of bias was low in all studies. The pooled treatment success, death, and adverse events of high-dose INH-containing therapy was 76.5% (95% CI: 70.9%–81.8%; I^2^: 92.03%), 7.1% (95% CI: 5.3%–9.1%; I^2^: 73.75%), and 61.1% (95% CI: 43.0%–77.8%; I^2^: 98.23%), respectively. The high-dose INH administration is associated with significantly higher treatment success (RR: 1.13, 95% CI: 1.04–1.22; *p* < 0.01) and a lower risk of death (RR: 0.45, 95% CI: 0.32–0.63; *p* < 0.01). However, in terms of other outcomes (such as adverse events, and culture conversion rate), no difference was observed between high-dose INH and other treatment options (all *p* > 0.05). In addition, no publication bias was observed.

**Conclusion:** In MDR-TB patients, high-dose INH administration is associated with a favorable outcome and acceptable adverse-event profile.

**Systematic review registration:** identifier CRD42023438080

## Introduction

Tuberculosis (TB) remains a serious public health issue and one of the leading worldwide causes of mortality. According to Global Tuberculosis Report 2023, there were an estimated 10.6 million incident cases of TB in 2022 worldwide and an estimated 1.30 million deaths (Global Tuberculosis Report, 2023). Remarkably, the COVID-19 has ceased the global progress against tuberculosis and influenced it severely, making the situation worse than initially thought (Patra et al., 2022). For example, the estimated number of deaths from TB increased between 2019 and 2021, and an estimated 1.6 million death was reported in 2021 by the World Health Organization (WHO) (Bagcchi, 2023).

Multidrug-resistant TB (MDR-TB), resistant to at least isoniazid (INH) and rifampin, is the most urgent concern to combat TB and challenging TB control efforts. In 2021, the estimated proportion of people with TB who had MDR/RR-TB was 3.6% (95% confidence interval [CI]: 2.7%–4.4%) among new cases and 18% (95% CI: 11%–26%) among those previously treated (Bagcchi, 2023). Most patients with MDR-TB carry a poor outcome. In general, its treatment success is low, with a pooled estimate of 60% in a previous meta-analysis (Bastos et al., 2017). Regarding children with MDR-TB, the situation may be better, approximately 78% of children had a successful treatment outcome at the end of therapy (Harausz et al., 2018a). The treatment of MDR-TB requires prolonged multidrug regimens that often include toxic and weakly effective medications. A meta-analysis demonstrated that some medications such as second-line injectable drugs (amikacin: 10.2% [95% CI: 6.3%–16.0%]; kanamycin: 7.5% [95% CI: 4.6%–11.9%]; capreomycin: 8·2% [95% CI: 6.3%–10.7%]), aminosalicylic acid (11.6%, 95% CI:7.1%–18.3%), and linezolid (14.1%, 95% CI: 9.9%–19.6%) had the high incidence of adverse events leading to permanent drug discontinuation (Lan et al., 2020). In addition, long-term physical sequelae such as respiratory (44.4%; 95% CI: 36.7%–52.1%), hearing (26.7%; 95% CI: 23.85%–29.7%), musculoskeletal (10.1%; 95% CI: 7.0%–13.2%), neurological (8.4%; 95% CI: 6.5%–10.3%), renal (8.1%: 95% CI: 6.3%–10.0%), hepatic (7.3%; 95% CI: 5.1%–9.4%), and visual sequelae (4.5%; 95% CI: 2.7%–6.3%) were common among survivors of MDR-TB (Akalu et al., 2023). Therefore, more efforts are required to improve outcomes by customizing treatment regimens.

Previously, a MDR-TB regimen containing high-dose INH is recommended by WHO (WHO, 2019). This recommendation is made under the assumption that some mutants (such as *inhA*) are associated with low-level INH resistance (Dominguez et al., 2023). Due to insufficient efficacy data, high-dose INH was removed from standard treatment recommendations in recent WHO guidance (Organization, 2020). However, high-dose INH is still indicated for children, patients without sufficient choice (due to adverse events, unavailable drug, and drug resistance, etc.), and special mutants (such as *inhA*, indicating low-level INH resistance) (Dooley et al., 2020; Rivière et al., 2020; Gausi et al., 2021; Dominguez et al., 2023). Interestingly, a study from India showed that the percentage of *inhA* mutation had an increasing trend during past years (Palani et al., 2022). Moreover, although high-dose INH is thought ineffective for strains with *katG* mutation, some evidence supported that higher doses of INH may have clinical efficacy, which is explained by the association between *katG* S315T mutation and intermediate level of INH resistance (Cambau et al., 2015; Lempens et al., 2018).

Although individualized treatment (according to minimum inhibitory concentration [MIC] and genotypic data) has been confirmed to be superior to standardized regimens, the latter remains the recommended therapy for MDR-TB in practice (Abidi et al., 2020; Rivière et al., 2020; Chang et al., 2021). It mainly because MIC and genotypic analysis are not available in many high-burden settings (Allix-Béguec et al., 2018; Davedow et al., 2022), which limits the access of individualized anti-TB treatment. The present study aimed to review current evidence and assess whether high-dose INH-containing therapy resulted in better treatment outcomes than other available regimens among patients diagnosed with MDR-TB. Although some studies have been conducted on WHO-endorsed shorter-course regimen (including high-dose INH) to discuss their benefits and drawbacks, no systematic reviews and meta-analyses have recently been published on this topic. Our study will provide evidence on efficiency and safety of MDR-TB treatment (containing high-dose INH) to inform the way forward to improve the outcomes of MDR-TB.

## Methods

### Search strategy

We conducted the systematic review and meta-analysis using the updated PRISMA guidelines (Page et al., 2021), and the protocol for this review has been registered in PROSPERO (CRD42023438080).

We searched the following databases for studies published from inception to 20 June 2023: PubMed, Embase, Scopus, Web of Science, CINAHL, the Cochrane Library, and ClinicalTrials.gov. The search terms included high-dose INH and MDR-TB. More details on search strategy were provided in Supplementary Material.

### Definitions and outcomes

The high-dose INH means daily dose of INH is > 300 mg/day or >5 mg/kg/day, which is higher than currently recommended (Nahid et al., 2016). Adverse and severe adverse events (SAEs) were recorded as reported in included studies. Our primary outcome was the proportion of treatment success, which is defined as a combination of cured and treatment completion. Secondary outcome included the proportion of adverse events (or SAEs), treatment unsuccess (death, lost-to-follow, and failure), sputum culture conversion at 2 months, 4 months, and 6 months.

### Inclusion and exclusion criteria

We included original research that investigated the treatment outcome of MDR-TB patients administrated with anti-TB therapy containing high-dose INH. Among them, at least one of the following outcomes was reported: treatment success (cure, completion), treatment unsuccess (death, failure, loss to follow-up), adverse events, and sputum culture conversion. The exclusion criteria included duplicates, reviews, book chapters, editorials, protocols, comments (or replies), ongoing clinical trials, irrelevant studies, insufficient data (such as lack of treatment outcome), small sample size (<10 cases), non-English literature, and non-peer reviewed articles.

### Study selection and data extraction

Briefly, two independent reviewers (ZM, CYL) screened the titles and abstracts for potential eligibility. Subsequently, full texts of eligible articles were retrieved, and eligibility criteria applied. Data extraction was done by two reviewers (ZM, CYL) independently. Any discrepancies between reviewers were resolved through referral to a third independent reviewer (LAM or YXB).

Extracted data included study characteristics (first author, year of publication, study design, district, and study period), patient characteristics (subjects, sample size, age, gender, HIV status, and history of anti-TB therapy), high-dose INH treatment (case number, dosage, duration, and other medications), standard treatment (control group; without INH), and outcomes (*n*; success, unsuccess, adverse, and sputum culture conversion).

### Quality assessment

The Newcastle-Ottawa Scale (NOS) (Stang, 2010) was used to assess the quality of observational studies, it assigns a maximum of 9 points based on three quality parameters: selection, comparability, and outcome, and the bias risk of each study is then rated as high (≤4), medium (5–6), and low (7–9), respectively. The quality of randomized controlled trials (RCTs) was evaluated using Jadad Scale (Clark et al., 1999), which consists of three fields: randomization, double blinding, and withdrawals and dropouts. Jadad scores range from 0 to 7, while trials scoring 4 or greater are considered to have a good quality.

### Statistical analysis

All statistical analyses were performed with STATA (Version, 15.0; Stata Corporation, College Station, TX). To evaluate treatment outcomes and adverse events, the estimates and corresponding 95% CIs were calculated depending on the heterogeneity (Q test and I^2^ statistics). If *p* < 0.05 or I^2^ > 50%, the heterogeneity is considered significant and a random-effect model was applied. Otherwise, a fixed-effect model was adopted. In addition, the outcomes stratified by possible influencing factors (such as study design, age, and high-dose INH duration) were also estimated. Relative risk (RR) is calculated by dividing the risk of an outcome (such as treatment success, and adverse events) in the exposed (high-dose INH) group by the risk in the unexposed (control) group. Publication bias was evaluated by Egger’s test. A *p*-value less than 0.05 was considered statistically significant.

## Results

### Literature selection

**FIGURE 1:**
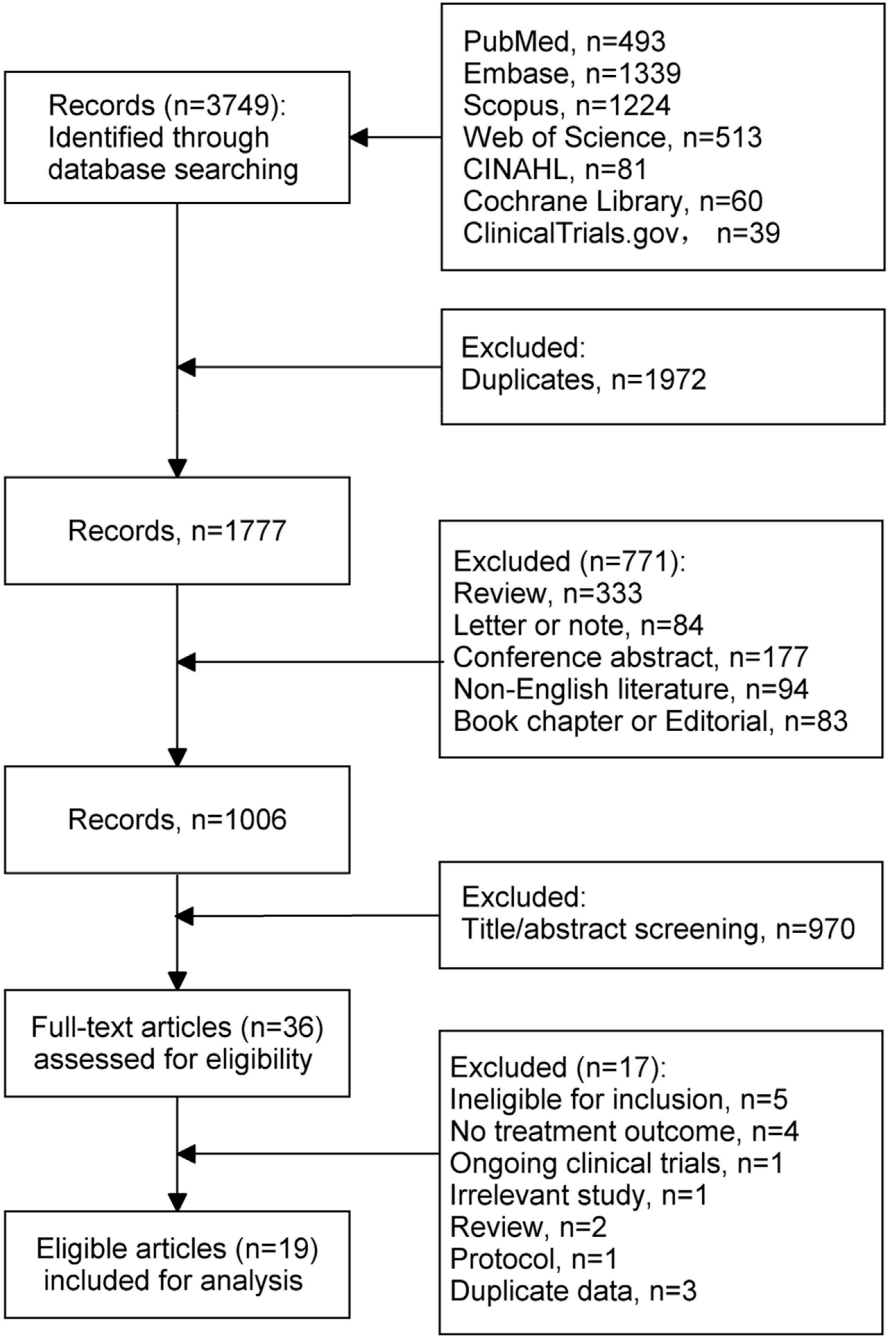
Literature selection process.

Figure 1 shows the flow chart of literature selection. Overall, 3,749 records were identified through search strategies. Of those, 1972 duplicates and 771 records that did not meet the objective criteria were excluded. After screening the titles and abstracts, 36 studies were assessed for eligibility. Of them, 17 were further excluded for the following reasons: ineligible for inclusion (*n* = 5; standard- or undefined-dose INH, *n* = 2; specific indications of INH, *n* = 2; non-MDR-TB patients, *n* = 1), no treatment outcome (*n* = 4), ongoing clinical trials (*n* = 1), irrelevant study (*n* = 1), review (*n* = 2), protocol (*n* = 1), duplicate data (*n* = 3). Finally, 19 studies were included for final analysis.

### Study characteristics

The characteristics of the 19 included studies are summarized in Table 1; Supplementary Table S1. Of the 19 studies, 2 were RCTs, 5 prospective cohort studies, and 12 retrospective cohort studies. Two studies included only children, and others (*n* = 18) included participants without age restriction. The sample size of included studies ranged from 26 to 1,006, with a total of 20 cohorts (*n* = 5,103). Of them, 3,728 were exposed to high-dose INH. Usually, high-dose INH was administrated during the intensive phase, except one study. In most studies (*n* = 17), high-dose INH was administrated for 4–6 months, and 3 studies used at least 6 months. Most studies (*n* = 12) were single-arm research.

**TABLE 1:** Baseline characteristics of eligible studies.

Study characteristics					Patient characteristics						Treatment			Outcomes			
No.	Author, year	District	Study design	Study period	Subjects	Sample size (*n*)	Age (y; Mean, or Median)	Male (*n*,%)	HIV+ (*n*,%)	Previous anti-TB therapy (*n*,%)	Exposed to high-dose INH (*n*,%)	High-dose INH duration	Control group	Definition of outcomes	Success (*n*,%)	Adverse events (*n*,%)	Culture conversion (*n*,%)
1	Katiyar et al. (2008)	India	RCT	2004.1–2005.12	Aged ≥18, Culture+, HIV-, MDR-TB	123	39.28	103, 83.7%	0	111, 90.2%	42, 34.1%	>6 montds	Yes	N/A	—	—	31, 73.8% (6 m)
2	Van Deun et al. (2010)	Bangladesh	PC	1997.5–2007.12	HIV-, MDR-TB	427	33.8	318, 74.5%	0	427, 100%	206, 48.2%	≤6 months	Yes	WHO	181, 87.86%	76, 36.89%	—
3	Piubello et al. (2014)	Niger	PC	2008–2010	MDR-TB	65	31	53, 81.5%	1, 1.7%	64, 98.5%	65, 100%	≤6 months	No	WHO	58, 89.23%	41, 63.07%	61, 98.38% (4 m); 62, 100% (6 m)
4	Trebucq et al. (2018)	West and Central Africa	PC	2013.1–2015.3	Aged ≥18, MDR-TB	1,006	34	667, 66.3%	200, 20%	872, 86.7%	1,006, 100%	≤6 months	No	WHO	821, 81.61%	897, 89.16%; 107, 10.63% (SAEs)	—
5	Harouna et al. (2019)	Niger	RC	2008.7–2013.9	Aged ≥20, MDR-TB	110	31	92, 84%	5, 5%	N/A	110, 100%	≤6 months	No	WHO	98, 89.09%	75, 68.18%	—
					Aged 1–19, MDR-TB	10	17	7, 70%	1, 10%		10, 100%	≤6 months	No		8, 80%	5, 50%	—
6	Walsh et al. (2019)	Haiti	RC	2009.1–2015.12	Aged ≥18, HIV-, MDR-TB	187	29	96, 51%	0	183, 98%	99, 53%	>6 months	Yes	WHO	88, 88.88%	—	—
7	Zhdanova et al. (2021)	Kyrgyzstan	RC	2016–2017	MDR/RR-TB	488	N/A	283, 58%	26, 5%	182, 37%	132, 27%	≤6 months	Yes	WHO	110, 83.33%	—	62, 46.96% (2 m); 79, 59.84% (4 m); 81, 61.36% (6 m)
8	Pirmahmadzoda et al. (2021)	Tajikistan	RC	2013.1–2019.7	Aged 0–17, MDR/RR-TB	60	13.6	20, 33%	1, 1.6%	10, 17%	7, 11.7%	≤6 months	Yes	WHO	7, 100%	—	—
9	Wahid et al. (2021)	Pakistan	RC	2018.1.1–2019.7.31	MDR-TB	313	33.7	156, 49.8%	N/A	219, 70%	313, 100%	≤6 months	No	WHO	262, 83.7%	—	221, 70.6% (2 m)
10	du Cros et al. (2021)	Uzbekistan	PC	2013.9.1–2015.3.31	MDR/RR-TB	128	30.1	61, 47.7%	0	30, 23.4%	128, 100%	≤6 months	No	WHO	92, 71.87%	100, 78.12%; 28, 21.87% (SAEs)	—
11	Trubnikov et al. (2021)	Uzbekistan	RC	2018.6–2019.9	Aged ≥15, MDR/RR-TB	95	45.6	67, 70.5%	11, 11.6%	49, 51.6%	95, 100%	≤6 months	No	WHO	63, 66.31%	38, 40%; 21, 22.10% (SAEs)	—
12	Mason et al. (2021)	Papua New Guinea	RC	2017.9–2018.9	Aged ≥15, RR-TB	26	N/A	15, 58%	5, 19%	N/A	26, 100%	≤6 months	No	WHO	10, 38.46%	10, 38.46% (SAEs)	—
13	Koirala et al. (2021)	Nepal	RC	2018.1–2019.12	Aged ≥15, MDR/RR-TB	301	35	216, 71.8%	13, 4.3%	172, 57.1%	301, 47.7%	≤6 months	No	France (IUATLD)	239, 79.4%	46, 15.28% (SAEs)	224, 90.68% (2 m); 224, 96.13% (4 m); 214, 97.71% (6 m)
14	Abubakar et al. (2022)	Pakistan	RC	2010.5.1–2017.6.30	XDR-TB	355	32.99	187, 52.7%	N/A	328, 92.3%	35, 9%	>6 months	Yes	WHO and NTP	8, 22.85%	—	—
15	Soeroto et al. (2022)	Indonesia	RC	2017.9–2020.12	Aged ≥18, MDR-TB	315	Unsuccessful: 40	177, 56.2%	0	271, 86%	315, 100%	≤6 months	No	WHO	202, 64.12%	—	197, 62.53% (2 m)
							Successful: 18										
16	Indarti et al. (2022)	Indonesia	RC	2016–2018	Aged ≥18, MDR-TB	99	48.97	69, 69.7%	44, 44.4%	93, 93.9%	65, 65.7%	≤6 months	Yes	WHO	23, 35.38%	—	—
17	Mleoh et al. (2023)	Tanzania	RC	2018.1–2020.8	MDR-TB	382	N/A	250, 65.4%	141, 36.9%	159, 41.6%	160, 41.9%	≤6 months	Yes	WHO	140, 87.5%	53, 33.12%	—
18	Kumari et al. (2023)	India	PC	2018.3–2020.2	Aged ≥15, MDR-TB	360	41	268, 74.4%	47, 13.1%	N/A	360, 100%	≤6 months	No	WHO	303, 84.16%	281, 78.05%	—
19	Nunn et al. (2019)	Ethiopia, Mongolia, South Africa, Vietnam	RCT	2012.7–2015.6	Aged ≥18, MDR-TB	253	N/A	151, 59.7%	85, 34%	235, 92.9%	253, 100%	≤6 months	No	N/A	193, 78.77%	136, 48.22% (SAEs)	145, 57.31% (2 m); 247, 97.62% (4 m); 252, 99.6% (6 m)

Abbreviations: RCT: randomized controlled trial; RC: retrospective cohort study; PC: prospective cohort study; MDR: multidrug-resistant; XDR: extensively drug-resistant; RR: rifampin-resistant; TB: tuberculosis; N/A: not available; HIV: human immunodeficiency virus; INH: isoniazid; SAEs: serious adverse events; WHO: world health organization; IUATLD: international union against tuberculosis and lung disease; NTP: National TB, control program.

### Quality assessment

The bias risk of included studies was assessed and detailed in Supplementary Tables S2, S3. For cohort studies, the NOS tool was used and the average score was 6.4 (out of 9). We used the Jadad scale to assess the RCTs and the average score was 4 (out of 7). All included studies were considered to have a low risk of bias.

### Treatment outcome

**FIGURE 2:**
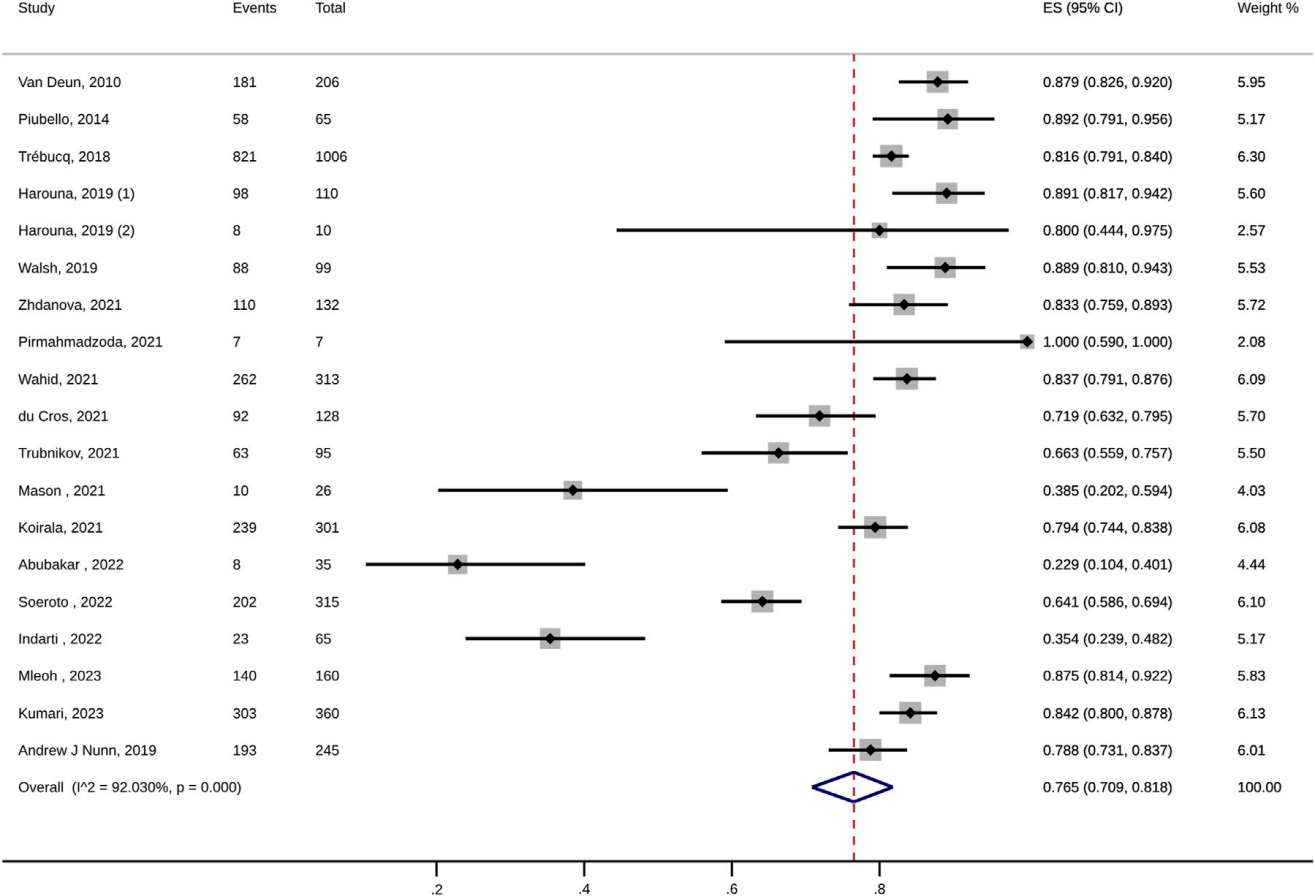
Forest plot of pooled treatment success for high-dose INH group.

For MDR-TB patients, the pooled treatment success rate of anti-TB therapy containing high-dose INH was 76.5% (95% CI: 70.9%–81.8%; I^2^: 92.03%; Figure 2; Table 2). In addition, no significant publication bias was detected (Egger’s test, *p* = 0.16). The death and treatment failure were also pooled and estimated at 7.1% (95% CI: 5.3%–9.1%; I^2^: 73.75%) and 4.9% (95% CI: 2.2%–8.5%; I^2^: 92.22%), respectively. The rates of culture conversion (at 2-month, 4-month, and 6-month) were 66.9% (95% CI: 51.7%–80.6%; I^2^: 96.78%; Egger’s test *p* = 0.53), 91.5 (95% CI: 73.8–99.9; I^2^: 97.32%; Egger’s test, *p* = 0.69), and 91.7% (95% CI: 73.7%–100%; I^2^: 97.43%; Egger’s test, *p* = 0.5), respectively (Table 2).

**TABLE 2:** Pooled estimates for outcomes of all MDR-TB patients treated with anti-TB therapy containing high-dose INH.

Treatment outcomes	Number of cohorts	Events/total (n/N)	Proportion^a^ (%, 95% CI)	I^2^ (%)	Egger’s test
Treatment success	19	2,906/3,678	76.5 (70.9–81.8)	92.03	0.16
Cure	10	1753/2,678	60.3 (49.6–70.7)	96.20	0.26
Completion	10	415/2,678	14.9 (6.5–25.7)	97.52	0.5
Treatment unsuccess	19	752/3,678	23.1 (17.6–29)	92.76	0.16
Death	16	291/3,537	7.1 (5.3–9.1)	73.75	0.63
Failure	14	171/3,227	4.9 (2.2–8.5)	92.22	0.23
Loss to follow-up	13	203/3,021	6.8 (3.1–11.5)	93.83	0.32
Culture conversion (2 m)	5	849/1,260	66.9 (51.7–80.6)	96.78	0.53
Culture conversion (4 m)	4	611/680	91.5 (73.8–99.9)	97.32	0.69
Culture conversion (6 m)	5	640/708	91.7 (73.7–100)	97.43	0.5
Adverse events	9	1,566/2,140	61.1 (43.0–77.8)	98.23	0.07
Severe adverse events	6	348/1838	24.6 (12.3–39.3)	97.18	0.32

^a^ Pooled estimates with 95% CI, was assessed using a random-effect model.

Compared with that of control group, the anti-TB therapy containing high-dose INH appears to have favorable outcomes (Supplementary Table S4; all *p* > 0.05), such as treatment success (79.6% vs. 69.3%), cure (60.4% vs. 57.7%), treatment completion (20.3% vs. 12.4%), treatment unsuccess (20.4% vs. 33.9%), death (4.5% vs. 12.8%), treatment failure (1.0% vs. 6.3%), loss to follow-up (11.7% vs. 17.8%), adverse events (34.6% vs. 44.0%), and sputum culture conversion a 6 months (64.9% vs. 53.5%).

**FIGURE 3:**
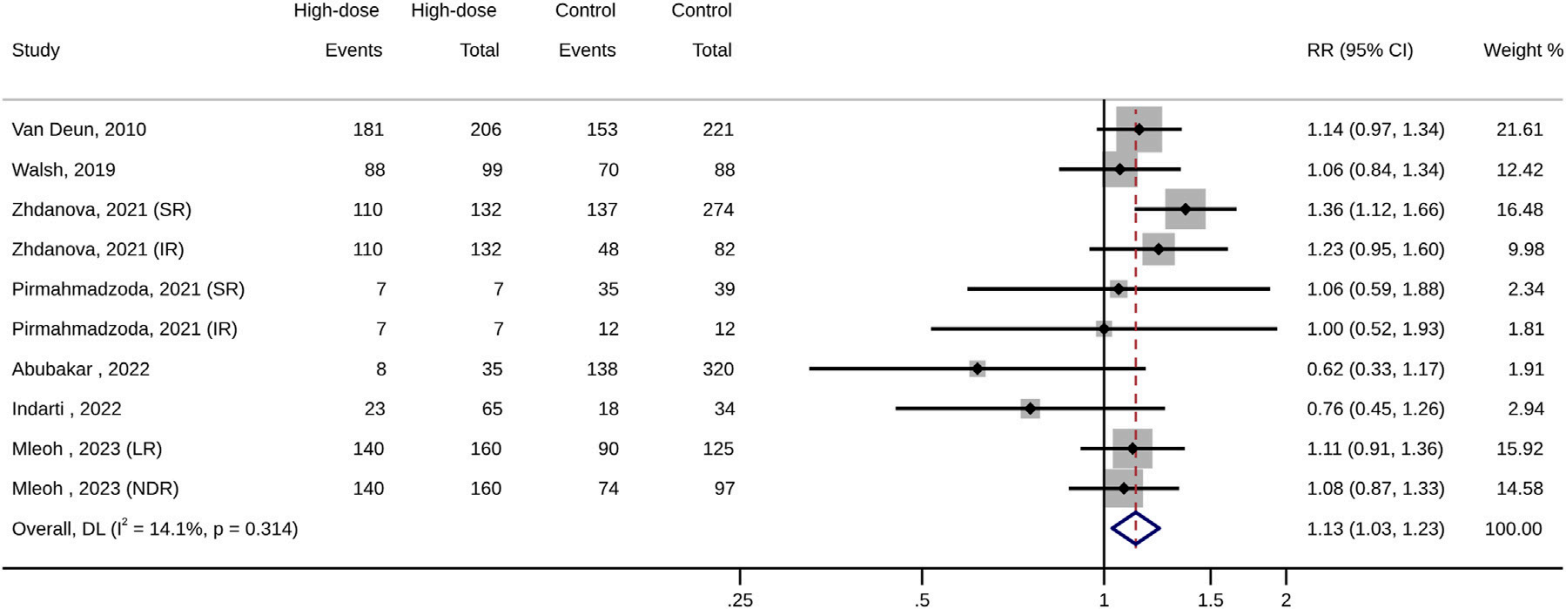
Forest plot of pooled risk ratios of treatment success (high-dose INH versus control groups).

For the management of patients with MDR-TB, the high-dose INH administration is associated with a higher treatment success rate (RR: 1.13, 95% CI: 1.04–1.22; *p* < 0.01; Figure 3; Table 3) and a lower risk of treatment unsuccess (RR: 0.63, 95% CI: 0.45–0.89; *p* = 0.01), including death (RR: 0.45, 95% CI: 0.32–0.63; *p* < 0.01). However, in terms of other outcomes (such as adverse events, and culture conversion rate), no difference were observed between high-dose INH and other treatment options (all *p* > 0.05; Table 3).

**TABLE 3:** Risk ratios of outcomes among high-dose INH versus control groups.

Treatment outcomes	Number of cohorts (*n*)	Case (*n*; high-dose INH/control groups)	Effects model (random)				Effects model (fixed)			
			Risk ratio (95%CI)	*p*-value	I^2^, %	Q test	Risk ratio (95%CI)	*p*-value	I^2^, %	Mantel-Haenszel Q
Treatment success	10	1,003/1,292	1.13 (1.03–1.23)	0.01	14.10	0.31	1.13 (1.04–1.22)	0.00	14.30	0.31
Cure	8	869/884	1.13 (0.99–1.29)	0.06	30.70	0.18	1.13 (1.03–1.25)	0.01	30.70	0.18
Completion	7	663/663	1.43 (1.11–1.84)	0.00	0.00	0.44	1.41 (1.09–1.81)	0.01	0.00	0.43
Treatment unsuccess	10	1,003/1,280	0.63 (0.45–0.89)	0.01	74.30	0.00	0.62 (0.53–0.73)	0.00	75.50	0.00
Death	6	855/833	0.55 (0.36–0.84)	0.01	23.30	0.26	0.45 (0.32–0.63)	0.00	39.80	0.14
Failure	4	535/611	0.37 (0.11–1.21)	0.10	56.50	0.08	0.31 (0.16–0.6)	0.00	59.00	0.06
Loss to follow-up	5	649/612	0.57 (0.31–1.07)	0.08	67.50	0.02	0.6 (0.44–0.8)	0.00	68.00	0.01
Culture conversion (2 m)	2	264/356	1.13 (0.92–1.4)	0.22	0.00	0.36	1.13 (0.92–1.4)	0.22	0.00	0.36
Culture conversion (4 m)	2	264/356	1.12 (0.93–1.34)	0.21	0.00	0.71	1.12 (0.93–1.34)	0.21	0.00	0.70
Culture conversion (6 m)	4	348/437	1.13 (0.97–1.32)	0.10	0.00	0.64	1.13 (0.97–1.32)	0.10	0.00	0.63
Adverse events	3	526/443	0.99 (0.53–1.83)	0.98	89.20	0.00	0.79 (0.67–0.94)	0.01	89.30	0.00

### Subgroup analyses

Subgroup analyses (Table 4) were performed, stratified by study design (prospective vs. retrospective), age (children vs. others), sample size (high-dose INH group; cases ≤50 vs. >50 cases), high-dose INH duration (≤6 vs. >6 months), control group (single arm study, yes/no), and definition of outcomes (WHO vs. others). The results of subgroup analyses showed that a large sample size of high-dose INH group (>50) was associated with heterogeneity and had a better treatment outcome (Table 4). Additionally, the single-arm design has a higher incidence of culture conversion at 6-month (Supplementary Table S5). Hence, study design of included studies remains a concern for the quality of our study. In contrast, to limit selection bias, a systematic review for high-dose INH is deserved our efforts.

**TABLE 4:** Subgroup analyses for the proportion of treatment success among MDR-TB patients treated with anti-TB therapy containing high-dose INH.

Variables	Study number (*n*)	Events/total (n/N)	Treatment success (%, 95% CI)	I^2^ (%)	Meta-regression analysis (*p*-value)
Study design					
Prospective cohort study	5	1,455/1765	83.1 (78.6–87.1)	75.36	0.26
Retrospective cohort study	13	1,258/1,668	72.6 (62.7–81.5)	93.66	
Subjects					
Adult/adult and children	18	2,891/3,661	75.6 (69.8–80.9)	92.83	0.69
Children	2	15/17	91.3 (70.7–100)	—	
Sample size (n, high-dose INH group)					
≤50	4	33/78	60.3 (24.6–91.3)	88.21	0.023^a^
>50	15	2,873/3,670	79.4 (74.5–83.8)	90.84	
High-dose isoniazid duration					
>6 months	2	96/134	74.5 (66.7–81.7)	—	0.40
≤6 months	17	2,810/3,544	78.1 (72.8–82.9)	90.56	
Control group					
Yes	7	557/704	74.8 (56.9–89.4)	95.47	0.74
No	12	2,349/2,974	77.7 (72.6–82.5)	88.15	
Definition of outcomes					
WHO	16	2,466/3,097	78.7 (72.7–84.1)	91.42	0.26
Others	3	440/581	74.4 (69.5–79)	—	

^a^ Adjusted R-squared = 57.99%.

### Adverse events

Among subjects administrated with high-dose INH-containing therapy, the rate of adverse events was pooled at 61.1% (95% CI: 43.0%–77.8%; I^2^: 98.23%; Egger’s test, *p* = 0.07; Table 2; Supplementary Table S6). Further analysis showed that the single-arm design contributed to the heterogeneity, and the design leads to a higher incidence of adverse events (Supplementary Table S7; 35.2% vs. 69.3%, *p* = 0.02). Gastrointestinal symptoms (such as vomiting, abdominal pain, anorexia, constipation, diarrhea, nausea, gastritis) were the most common reported events with a pooled estimate of 51.5% (95% CI: 43.5%–59.3%; I^2^: 82.49%; Table 5). Besides, ototoxicity, neurotoxicity, cardiotoxicity, hepatotoxicity, nephrotoxicity arthralgia, endocrine disorders, and dermatologic systems were also common and their prevalence were estimated at 20.5% (95% CI: 6.8%–38.5%; I^2^: 97.53%), 12.4% (95% CI: 5.2%–22.0%; I^2^: 93.66%), 12.2% (95% CI: 0%–39.1%; I^2^: 98.87%), 10.8% (95% CI: 6.2%–16.4%; I^2^: 81.23%), 8.4% (95% CI: 3.3%–15.5%; I^2^: 90.88%), 7.1% (95% CI: 0.5%–19.0%; I^2^: 84.72%), and 5.8% (95% CI: 2.5%–10.1%; I^2^: 57.94%), respectively. The SAEs of therapy containing high-dose INH were reported in 6 studies and its prevalence was estimated at 24.6% (95% CI: 12.3%–39.3%; I^2^: 97.18%; Table 5). Hepatotoxicity was the most common SAE (42.4%, 95% CI: 13.6%–74%; I^2^: 92.56%), followed by ototoxicity (29.2%, 95% CI: 9.5%–53.8%; I^2^: 87.23%), cardiotoxicity (15.6%, 95% CI: 1.5%–38.1%; I^2^: 87.77%), and electrolyte disturbance (3.0%, 95% CI: 0%–10.2%; I^2^: 58.32%).

**TABLE 5:** Pooled estimates for the proportion of adverse events among the high-dose INH group, stratified by organ systems.

Types	Adverse events				Severe adverse events			
	Number of cohorts (*n*)	Number of events (*n*)	Proportion (%, 95% CI)	I^2^ (%)	Number of cohorts (n)	Number of events (n)	Proportion (%, 95% CI)	I^2^ (%)
Gastrointestinal symptom	9	920	51.5 (43.5–59.3)	82.49	0	0	—	—
Ototoxicity	9	535	20.5 (6.8–38.5)	97.53	3	52	29.2 (9.5–53.8)	87.23
Neurotoxicity	7	331	12.4 (5.2–22.0)	93.66	1	1	—	—
Hepatotoxicity	7	518	12.2 (0–39.1)	98.87	3	61	42.4 (13.6–74.0)	92.56
Nephrotoxicity	5	199	10.8 (6.2–16.4)	81.23	1	2	—	—
Arthralgia	6	224	8.4 (3.3–15.5)	90.88	0	0	—	—
Endocrine disorders	3	14	7.1 (0.5–19)	84.72	1	3	—	—
Dermatologic symptoms	5	27	5.8 (2.5–10.1)	57.94	1	2	—	—
Cardiotoxicity	2	6	3.5 (0.8–7.6)	—	3	45	15.6 (1.5–38.1)	87.77
Psychiatric disorders	4	7	2.4 (0.6–4.9)	0	1	2	—	—
Visual impairment	4	6	2.2 (0.5–4.8)	0	1	2	—	—
Hematological disorders	2	8	2.0 (0.8–3.8)	—	0	0	—	—
Electrolyte disturbance	2	2	1.2 (0–3.9)	—	3	7	3.0 (0–10.2)	58.32
Others	2	49	12.5 (9.3–20.0)	—	1	2	—	—

## Discussion

MDR-TB is a serious threat to the effort to control TB (Dean et al., 2022). Its treatment is extremely difficult and a prolonged therapy is required (Migliori et al., 2020). This leads to a poor adherence and significant adverse events (Okethwangu et al., 2019; Lan et al., 2020; Bothamley, 2022). Hence, a shorter and effective treatment regime is very essential. In recent years, a number of new therapeutics aimed at tackling MDR-TB have emerged. In particular, there has been increasing interest in assessing the efficacy of short-course regimens (Dookie et al., 2022). Novel treatment, such as delamanid, bedaquiline, and linezolid, has been introduced for MDR-TB treatment. Data suggests that the treatment success rates of therapies containing delamanid, bedaquiline, low-dose linezolid, and a combination of delamanid and bedaquiline were 80.9% (95% CI: 72.6%–87.2%) (Nasiri et al., 2022), 74.7% (95% CI: 69.8%–79.0%) (Hatami et al., 2022), 91% (Mase et al., 2022) and 75.2% (95% CI: 68.1%–81.1%) (Nasiri et al., 2022), respectively. However, the drug availability, cost, and drug susceptibility testing capacity of these novel regimes remain a concern (Günther et al., 2023; Xu et al., 2023).

In the meta-analysis, we showed that high-dose INH administration was associated with an increased rate of treatment outcome (79.6% vs. 69.3%; RR: 1.13, 95% CI: 1.04–1.22; *p* < 0.01) and a decreased rate of death (4.5% vs. 12.8%; RR: 0.45, 95% CI: 0.32–0.63; *p* < 0.01). Regarding the results of other outcomes (such as adverse events), our findings supported that anti-TB therapy containing high-dose INH is superior to standard regimes, although non-significant statistical difference were observed between the two groups. To our knowledge, this is the first meta-analysis of high-dose INH for MDR-TB treatment, which provides confirmed evidence to tailor the MDR-TB treatment guidelines.

In this study, we found that MDR-TB treatment containing high-dose INH achieved good treatment success, with a pooled estimate of 76.5% (95% CI: 70.9%–81.8%). Previously published studies of similar WHO-endorsed shorter-course regimen (including high-dose INH) have reported end-of-treatment success between 80.0% and 83.0%, which is similar to our outcome (Ahmad Khan et al., 2017; Abidi et al., 2020). In addition, data from a South African cohort of INH-resistant TB suggested that high-dose INH administration leads to greater odds of successful outcome, even in those with *katG* mutations (Jacobson et al., 2011). A previous meta-analysis of childhood MDR-TB demonstrated that high-dose INH was associated with treatment success (adjusted OR: 5.9, 95% CI 1.7%–20.5%; *p* = 0.007), however, the majority of included patients came from a same site, Cape Town (Harausz et al., 2018b).

In a previous meta-analysis, the proportion of death outcome in individuals with MDR-TB was 14% (Ahmad et al., 2018). This is consistent with our findings, and the pooled death rate of control group was 12.8%. In contrast, fewer patients treated with the high-dose INH experienced death, with a pooled rate of 4.5%. However, a previous meta-analysis demonstrated that patients treated with the shorter regimen experienced more death (shorter 7.6% vs. longer 4.6%) (Abidi et al., 2020), which is in not in line with our findings. This may be explained by that high-dose INH is not necessary in case of the WHO shorter-course recommendation and no restriction is posed on the regimes (shorter or longer duration) in our study.

Among subjects administrated with high-dose INH, the prevalence of adverse events was estimated at 61.1% (95% CI: 43.0%–77.8%), and the major adverse events identified was gastrointestinal symptoms (51.5%, 95% CI: 43.5%–59.3%). However, other adverse effects were not uncommon. It was demonstrated that the percentage of adverse events were not significantly different between high-dose INH and control groups. Additionally, the prevalence of SAEs was estimated at 24.6% (95% CI: 12.3%–39.3%), and hepatotoxicity (42.4%, 95% CI: 13.6%–74%) was the most common SAE. It seems like that administration of high-dose INH could not increase the occurrence of adverse events. This indicates that the high-dose INH is well tolerable without significant SAEs. Similar prevalence (58.9%) of adverse events has been reported for linezolid-containing regimes during MDR-TB treatment (Sotgiu et al., 2012). Although adverse events are common in our study, these events are not mainly associated with high doses of INH. Because hepatotoxicity is known as the most common side effect of INH administration (Tweed et al., 2018; Hamada et al., 2020; Sterling et al., 2020; Melnychuk et al., 2023). Remarkably, considering the distribution of SAEs (mainly hepatotoxicity), the SAE is associated with the administration of INH. Therefore, a caution is required when using high-dose INH. In the future, better data would be needed for further analysis, which optimizes INH dosage strategy to balance favorable outcomes but minimizes risk of SAEs.

Our study has several strengths. First, our finding contributes to the ongoing debate about the effectiveness of the high-dose INH for MDR-TB treatment, which has not been fully addressed. Second, the analysis included a sufficient number of studies (*n* = 19) and a relatively large sample (5,103 patients), selection and confounding bias will be reduced by aggregated data. Third, the data, from a diversity of settings, has improved the generalizability and precision of the findings. Fourth, although increasing the drug dosage could lead to an increase in costs, it has substantial benefits for MDR-TB patients. Increasing the dose may even be less expensive in the long term, as more patients will have adequate exposure to the drug, resulting in more treatment success, fewer death, and acceptable SAEs. Lastly, this study is cost-saving, if such study is undertaken prospectively, the cost will be too great.

Although our study updated evidence on INH efficacy, there are also some limitations to this study. First, although treatment effect was estimated for high-dose INH-containing therapy, the analysis could not isolate the benefit of a single drug. Confounding variables may be present, and the finding should be interpreted with caution. Second, there was a high proportion of retrospective studies (*n* = 12). This increases the likelihood of reporting and selection bias. Third, there was significant clinical heterogeneity among the included studies, the heterogeneity was associated with study design. Hence, inferences should be made with caution. Fourth, owing to limited clinical data, it is challenging to assess the impact of clinical characteristics such as INH dosage, INH duration, and dose-response relationship of SAEs. Fifth, it does not evaluate adherence to treatment regimens containing high-dose INH, an important outcome determinant. At last, the existing evidence suggests that high-dose INH is effective in the presence of the *inhA* gene mutation and in absence of the *katG* mutation (Wasserman and Furin, 2020). Therefore, if the indication for INH resistance is introduced for the high-dose INH use, a better outcome would be archived.

## Conclusion

Our finding supports an excellent efficacy in the prescription of high-dose INH for treatment of MDR-TB, and high-dose INH administration is associated with a favorable outcome and acceptable adverse-event profile. In addition, a shorter INH-containing regime (less than 6 months) seems to be satisfactory, as it has a short duration while not compromising efficacy. However, further data are needed to analyze the impact of higher doses of INH on longer-term outcomes, such as disease relapse, as well as to investigate its role for specific subpopulations, such as patients with HIV (+) or *inhA* mutants.

## Data Availability

The original contributions presented in the study are included in the article/Supplementary Material, further inquiries can be directed to the corresponding authors.

## Abbreviations

TB, Tuberculosis; WHO, World Health Organization; MDR-TB, multidrug-resistant tuberculosis; INH, isoniazid; CI, Confidence interval; MIC, minimum inhibitory concentration; SAE, adverse and severe adverse event; NOS, Newcastle-Ottawa Scale; RCT, randomized controlled trial; RR, relative risk.
