# Supplementary material for: The efficacy and safety of high-dose isoniazid-containing therapy for multidrug-resistant tuberculosis: a systematic review and meta-analysis
Source: Front Pharmacol. 2024 Jan 8;14:1331371. doi: 10.3389/fphar.2023.1331371 (PMC10800833; doi:10.3389/fphar.2023.1331371)
Supplement: Supplementary file 1 [file DataSheet1.zip › Original data for STATA analysis (proportions)- high-dose INH group.DOCX]

| Supplementary materials- Original data for STATA analysis (proportions)- high-dose INH group. | | | | | | | | | | | | | | | | | | | | | | | | | | | | | | | |
| --- | --- | --- | --- | --- | --- | --- | --- | --- | --- | --- | --- | --- | --- | --- | --- | --- | --- | --- | --- | --- | --- | --- | --- | --- | --- | --- | --- | --- | --- | --- | --- |
| No. | Author, year | Study design | Cases (n) | Population | High-dose INH duration | Control group | Definition of outcomes | Success | | Cure | | Completion | | Unsuccess | | Death | | Failure | | LTFU | | Adverse events | | Severe adverse events | | Culture conversion (2m) | | Culture conversion (4m) | | Culture conversion (6m) | |
|  |  |  |  |  |  |  |  | Events (n) | Total (n) | Events (n) | Total (n) | Events (n) | Total (n) | Events (n) | Total (n) | Events (n) | Total (n) | Events (n) | Total (n) | Events (n) | Total (n) | Events (n) | Total (n) | Events (n) | Total (n) | Events (n) | Total (n) | Events (n) | Total (n) | Events (n) | Total (n) |
| 1 | Katiyar, 2008 | RCT | 42 | adult/adult and children | > 6 months | Yes | N/A |  |  |  |  |  |  |  |  |  |  |  |  |  |  |  |  |  |  |  |  |  |  | 31 | 42 |
| 2 | Van Deun, 2010 | PC | 206 | adult/adult and children | ≤ 6 months | Yes | WHO | 181 | 206 | 170 | 206 | 1 | 206 | 25 | 206 | 11 | 206 | 1 | 206 |  |  | 76 | 206 |  |  |  |  |  |  |  |  |
| 3 | Piubello, 2014 | PC | 65 | adult/adult and children | ≤ 6 months | No | WHO | 58 | 65 |  |  |  |  | 7 | 65 | 6 | 65 |  |  |  |  | 41 | 65 |  |  |  |  | 61 | 62 | 62 | 62 |
| 4 | Trébucq, 2018 | PC | 1006 | adult/adult and children | ≤ 6 months | No | WHO | 821 | 1006 | 728 | 1006 | 93 | 1006 | 185 | 1006 | 78 | 1006 | 59 | 1006 | 48 | 1006 | 897 | 1006 | 107 | 1006 |  |  |  |  |  |  |
| 5.1 | Harouna, 2019 (1) | RC | 110 | adult/adult and children | ≤ 6 months | No | WHO | 98 | 110 |  |  |  |  | 12 | 110 | 9 | 110 | 1 | 110 | 2 | 110 | 75 | 110 |  |  |  |  |  |  |  |  |
| 5.2 | Harouna, 2019 (2) | RC | 10 | children | ≤ 6 months | No | WHO | 8 | 10 |  |  |  |  | 2 | 10 | 1 | 10 | 1 | 10 | 0 | 10 | 5 | 10 |  |  |  |  |  |  |  |  |
| 6 | Walsh, 2019 | RC | 99 | adult/adult and children | > 6 months | Yes | WHO | 88 | 99 |  |  |  |  | 11 | 99 |  |  |  |  |  |  |  |  |  |  |  |  |  |  |  |  |
| 7 | Zhdanova, 2021 | RC | 132 | adult/adult and children | ≤ 6 months | Yes | WHO | 110 | 132 | 74 | 132 | 36 | 132 | 22 | 132 | 0 | 132 | 4 | 132 | 18 | 132 |  |  |  |  | 62 | 132 | 79 | 132 | 81 | 132 |
| 8 | Pirmahmadzoda, 2021 | RC | 7 | children | ≤ 6 months | Yes | WHO | 7 | 7 | 2 | 7 | 5 | 7 | 0 | 7 |  |  |  |  |  |  |  |  |  |  |  |  |  |  |  |  |
| 9 | Wahid, 2021 | RC | 313 | adult/adult and children | ≤ 6 months | No | WHO | 262 | 313 | 250 | 313 | 12 | 313 | 51 | 313 | 31 | 313 | 4 | 313 | 16 | 313 |  |  |  |  | 221 | 313 |  |  |  |  |
| 10 | du Cros, 2021 | PC | 128 | adult/adult and children | ≤ 6 months | No | WHO | 92 | 128 | 55 | 128 | 37 | 128 | 36 | 128 | 2 | 128 | 22 | 128 | 12 | 128 | 100 | 128 | 28 | 128 |  |  |  |  |  |  |
| 11 | Trubnikov, 2021 | RC | 95 | adult/adult and children | ≤ 6 months | No | WHO | 63 | 95 |  |  |  |  | 32 | 95 | 7 | 95 | 17 | 95 | 5 | 95 | 38 | 95 | 21 | 95 |  |  |  |  |  |  |
| 12 | Mason , 2021 | RC | 26 | adult/adult and children | ≤ 6 months | No | WHO | 10 | 26 |  |  |  |  | 16 | 26 | 2 | 26 | 12 | 26 | 2 | 26 |  |  | 10 | 26 |  |  |  |  |  |  |
| 13 | Koirala, 2021 | RC | 301 | adult/adult and children | ≤ 6 months | No | France (IUATLD) | 239 | 301 | 177 | 301 | 62 | 301 | 62 | 301 | 36 | 301 | 16 | 301 | 8 | 301 |  |  | 46 | 301 | 224 | 247 | 224 | 233 | 214 | 219 |
| 14 | Abubakar , 2022 | RC | 35 | adult/adult and children | > 6 months | Yes | WHO and NTP | 8 | 35 |  |  |  |  | 27 | 35 |  |  |  |  |  |  |  |  |  |  |  |  |  |  |  |  |
| 15 | Soeroto , 2022 | RC | 315 | adult/adult and children | ≤ 6 months | No | WHO | 202 | 315 |  |  |  |  | 113 | 315 | 27 | 315 | 30 | 315 | 56 | 315 |  |  |  |  | 197 | 315 |  |  |  |  |
| 16 | Indarti , 2022 | RC | 65 | adult/adult and children | ≤ 6 months | Yes | WHO | 23 | 65 | 23 | 65 | 0 | 65 | 42 | 65 | 6 | 65 | 2 | 65 | 32 | 65 |  |  |  |  |  |  |  |  |  |  |
| 17 | Mleoh , 2023 | RC | 160 | adult/adult and children | ≤ 6 months | Yes | WHO | 140 | 160 | 121 | 160 | 19 | 160 | 20 | 160 | 18 | 160 | 0 | 160 | 2 | 160 | 53 | 160 |  |  |  |  |  |  |  |  |
| 18 | Kumari, 2023 | PC | 360 | adult/adult and children | ≤ 6 months | No | WHO | 303 | 360 | 153 | 360 | 150 | 360 | 37 | 360 | 33 | 360 | 2 | 360 | 2 | 360 | 281 | 360 |  |  |  |  |  |  |  |  |
| 19 | Andrew J Nunn, 2019 | RCT | 253 | adult/adult and children | ≤ 6 months | No | N/A | 193 | 245 |  |  |  |  | 52 | 245 | 24 | 245 |  |  |  |  |  |  | 136 | 282 | 145 | 253 | 247 | 253 | 252 | 253 |
| Abbreviations: SD: standard dosage; SR: standard regimen; LR: longer regimen; NDR: new drug regimen; IR: individualized regimen; N/A: not available; RCT: randomized controlled trial; RC: retrospective cohort study; PC: prospective cohort study; WHO: World Health Organization; IUATLD: International Union Against Tuberculosis and Lung Disease; NTP: National TB Control Program | | | | | | | | | | | | | | | | | | | | | | | | | | | | | | | |
