# Supplementary material for: The efficacy and safety of high-dose isoniazid-containing therapy for multidrug-resistant tuberculosis: a systematic review and meta-analysis
Source: Front Pharmacol. 2024 Jan 8;14:1331371. doi: 10.3389/fphar.2023.1331371 (PMC10800833; doi:10.3389/fphar.2023.1331371)
Supplement: Supplementary file 1 [file DataSheet1.zip › Original data for STATA analysis (risk ratios)- high-dose INH group.DOCX]

| Supplementary materials- Original data for STATA analysis (risk ratios)- high-dose INH group. | | | | | | | | | | | | | | | | | | | | | | | | | | | | | | | | | | | | | | | | | | | | | | |  |  |  |  |
| --- | --- | --- | --- | --- | --- | --- | --- | --- | --- | --- | --- | --- | --- | --- | --- | --- | --- | --- | --- | --- | --- | --- | --- | --- | --- | --- | --- | --- | --- | --- | --- | --- | --- | --- | --- | --- | --- | --- | --- | --- | --- | --- | --- | --- | --- | --- | --- | --- | --- | --- |
| No. | Study | Study design | Population | High-dose INH duration | Control group | Definition of outcomes | Success | | | | Cure | | | | Completion | | | | Unsuccess | | | | Death | | | | Failure | | | | LTFU | | | | Culture conversion (2m) | | | | Culture conversion (4m) | | | | Culture conversion (6m) | | | | Adverse events | | | |
|  |  |  |  |  |  |  | High-dose INH group | | Control group | | High-dose INH group | | Control group | | High-dose INH group | | Control group | | High-dose INH group | | Control group | | High-dose INH group | | Control group | | High-dose INH group | | Control group | | High-dose INH group | | Control group | | High-dose INH group | | Control group | | High-dose INH group | | Control group | | High-dose INH group | | Control group | | High-dose INH group | | Control group | |
|  |  |  |  |  |  |  | Events (n) | Total (n) | Events (n) | Total (n) | Events (n) | Total (n) | Events (n) | Total (n) | Events (n) | Total (n) | Events (n) | Total (n) | Events (n) | Total (n) | Events (n) | Total (n) | Events (n) | Total (n) | Events (n) | Total (n) | Events (n) | Total (n) | Events (n) | Total (n) | Events (n) | Total (n) | Events (n) | Total (n) | Events (n) | Total (n) | Events (n) | Total (n) | Events (n) | Total (n) | Events (n) | Total (n) | Events (n) | Total (n) | Events (n) | Total (n) | Events (n) | Total (n) | Events (n) | Total (n) |
| 1.1 | Katiyar, 2008 (SD) | RCT | adult | > 6 months | Yes | N/A |  |  |  |  |  |  |  |  |  |  |  |  |  |  |  |  |  |  |  |  |  |  |  |  |  |  |  |  | 62 | 132 | 96 | 274 |  |  |  |  | 31 | 42 | 18 | 40 |  |  |  |  |
| 1.2 | Katiyar, 2008 (placebo) |  |  |  |  |  |  |  |  |  |  |  |  |  |  |  |  |  |  |  |  |  |  |  |  |  |  |  |  |  |  |  |  |  | 62 | 132 | 38 | 82 |  |  |  |  | 31 | 42 | 20 | 41 |  |  |  |  |
| 2 | Van Deun, 2010 | PC | adult and children | ≤ 6 months | Yes | WHO | 181 | 206 | 153 | 221 | 170 | 206 | 153 | 221 |  |  |  |  | 25 | 206 | 68 | 221 | 11 | 206 | 22 | 221 | 1 | 206 | 16 | 221 |  |  |  |  |  |  |  |  |  |  |  |  |  |  |  |  | 76 | 206 | 187 | 221 |
| 3 | Piubello, 2014 | PC | adult | ≤ 6 months | No | WHO | 58 | 65 |  |  |  |  |  |  |  |  |  |  | 7 | 65 |  |  | 6 | 65 |  |  |  |  |  |  |  |  |  |  |  |  |  |  |  |  |  |  |  |  |  |  |  |  |  |  |
| 4 | Trébucq, 2018 | PC | adult | ≤ 6 months | No | WHO | 821 | 1006 |  |  |  |  |  |  |  |  |  |  | 185 | 1006 |  |  | 78 | 1006 |  |  | 59 | 1006 |  |  | 48 | 1006 |  |  |  |  |  |  |  |  |  |  |  |  |  |  |  |  |  |  |
| 5.1 | Harouna, 2019 (1) | RC | adult | ≤ 6 months | No | WHO | 98 | 110 |  |  |  |  |  |  |  |  |  |  | 12 | 110 |  |  | 9 | 110 |  |  | 1 | 110 |  |  | 2 | 110 |  |  |  |  |  |  |  |  |  |  |  |  |  |  |  |  |  |  |
| 5.2 | Harouna, 2019 (2) | RC | children | ≤ 6 months | No | WHO | 8 | 10 |  |  |  |  |  |  |  |  |  |  | 2 | 10 |  |  | 1 | 10 |  |  | 1 | 10 |  |  | 0 | 10 |  |  |  |  |  |  |  |  |  |  |  |  |  |  |  |  |  |  |
| 6 | Walsh, 2019 | RC | adult | > 6 months | Yes | WHO | 88 | 99 | 70 | 88 |  |  |  |  |  |  |  |  | 11 | 99 | 18 | 88 |  |  |  |  |  |  |  |  |  |  |  |  |  |  |  |  |  |  |  |  |  |  |  |  |  |  |  |  |
| 7.1 | Zhdanova, 2021 (SR) | RC | adult and children | ≤ 6 months | Yes | WHO | 110 | 132 | 137 | 274 | 74 | 132 | 89 | 274 | 36 | 132 | 48 | 274 | 22 | 132 | 137 | 274 | 0 | 132 | 36 | 274 | 4 | 132 | 8 | 274 | 18 | 132 | 93 | 274 |  |  |  |  | 79 | 132 | 132 | 274 | 81 | 132 | 137 | 274 |  |  |  |  |
| 7.2 | Zhdanova, 2021 (IR) | RC | adult and children | ≤ 6 months | Yes | WHO | 110 | 132 | 48 | 82 | 74 | 132 | 29 | 82 | 36 | 132 | 19 | 82 | 22 | 132 | 34 | 82 | 0 | 132 | 4 | 82 | 4 | 132 | 12 | 82 | 18 | 132 | 18 | 82 |  |  |  |  | 79 | 132 | 44 | 82 | 81 | 132 | 50 | 82 |  |  |  |  |
| 8.1 | Pirmahmadzoda, 2021 (SR) | RC | children | ≤ 6 months | Yes | WHO | 7 | 7 | 35 | 39 | 2 | 7 | 30 | 39 | 5 | 7 | 5 | 39 | 0 | 7 | 4 | 39 |  |  |  |  |  |  |  |  |  |  |  |  |  |  |  |  |  |  |  |  |  |  |  |  |  |  |  |  |
| 8.2 | Pirmahmadzoda, 2021 (IR) | RC | children | ≤ 6 months | Yes | WHO | 7 | 7 | 12 | 12 | 2 | 7 | 8 | 12 | 5 | 7 | 4 | 12 | 0 | 7 | 0 | 0 |  |  |  |  |  |  |  |  |  |  |  |  |  |  |  |  |  |  |  |  |  |  |  |  |  |  |  |  |
| 9 | Wahid, 2021 | RC | adult and children | ≤ 6 months | No | WHO | 262 | 313 |  |  |  |  |  |  |  |  |  |  | 51 | 313 |  |  | 31 | 313 |  |  | 4 | 313 |  |  | 16 | 313 |  |  |  |  |  |  |  |  |  |  |  |  |  |  |  |  |  |  |
| 10 | du Cros, 2021 | PC | adult and children | ≤ 6 months | No | WHO | 92 | 128 |  |  |  |  |  |  |  |  |  |  | 36 | 128 |  |  | 2 | 128 |  |  | 22 | 128 |  |  | 12 | 128 |  |  |  |  |  |  |  |  |  |  |  |  |  |  |  |  |  |  |
| 11 | Trubnikov, 2021 | RC | adult | ≤ 6 months | No | WHO | 63 | 95 |  |  |  |  |  |  |  |  |  |  | 32 | 95 |  |  | 7 | 95 |  |  | 17 | 95 |  |  | 5 | 95 |  |  |  |  |  |  |  |  |  |  |  |  |  |  |  |  |  |  |
| 12 | Mason , 2021 | RC | adult | ≤ 6 months | No | WHO | 10 | 26 |  |  |  |  |  |  |  |  |  |  | 16 | 26 |  |  | 2 | 26 |  |  | 12 | 26 |  |  | 2 | 26 |  |  |  |  |  |  |  |  |  |  |  |  |  |  |  |  |  |  |
| 13 | Koirala, 2021 | RC | adult | ≤ 6 months | No | France (IUATLD) | 239 | 301 |  |  |  |  |  |  |  |  |  |  | 62 | 301 |  |  | 36 | 301 |  |  | 16 | 301 |  |  | 8 | 301 |  |  |  |  |  |  |  |  |  |  |  |  |  |  |  |  |  |  |
| 14 | Abubakar , 2022 | RC | adult and children | > 6 months | Yes | WHO and NTP | 8 | 35 | 138 | 320 |  |  |  |  |  |  |  |  | 27 | 35 | 182 | 320 |  |  |  |  |  |  |  |  |  |  |  |  |  |  |  |  |  |  |  |  |  |  |  |  |  |  |  |  |
| 15 | Soeroto , 2022 | RC | adult | ≤ 6 months | No | WHO | 202 | 315 |  |  |  |  |  |  |  |  |  |  | 113 | 315 |  |  | 27 | 315 |  |  | 30 | 315 |  |  | 56 | 315 |  |  |  |  |  |  |  |  |  |  |  |  |  |  |  |  |  |  |
| 16 | Indarti , 2022 | RC | adult | ≤ 6 months | Yes | WHO | 23 | 65 | 18 | 34 | 23 | 65 | 17 | 34 | 0 | 65 | 1 | 34 | 42 | 65 | 16 | 34 | 6 | 65 | 4 | 34 | 2 | 65 | 1 | 34 | 32 | 65 | 10 | 34 |  |  |  |  |  |  |  |  |  |  |  |  |  |  |  |  |
| 17.1 | Mleoh , 2023 (LR) | RC | adult and children | ≤ 6 months | Yes | WHO | 140 | 160 | 90 | 125 | 121 | 160 | 79 | 125 | 19 | 160 | 11 | 125 | 20 | 160 | 35 | 125 | 18 | 160 | 26 | 125 |  |  |  |  | 2 | 160 | 9 | 125 |  |  |  |  |  |  |  |  |  |  |  |  | 53 | 160 | 35 | 125 |
| 17.2 | Mleoh , 2023 (NDR) | RC | adult and children | ≤ 6 months | Yes | WHO | 140 | 160 | 74 | 97 | 121 | 160 | 68 | 97 | 19 | 160 | 6 | 97 | 20 | 160 | 23 | 97 | 18 | 160 | 18 | 97 |  |  |  |  | 2 | 160 | 5 | 97 |  |  |  |  |  |  |  |  |  |  |  |  | 53 | 160 | 18 | 97 |
| 18 | Kumari, 2023 | PC | adult | ≤ 6 months | No | WHO | 303 | 360 |  |  |  |  |  |  |  |  |  |  | 37 | 360 |  |  | 33 | 360 |  |  | 2 | 360 |  |  | 2 | 360 |  |  |  |  |  |  |  |  |  |  |  |  |  |  |  |  |  |  |
| 19 | Andrew J Nunn, 2019 | RCT | adult | ≤ 6 months | No | N/A | 193 | 245 |  |  |  |  |  |  |  |  |  |  | 52 | 245 |  |  | 24 | 245 |  |  |  |  |  |  |  |  |  |  |  |  |  |  |  |  |  |  |  |  |  |  |  |  |  |  |
| Abbreviations: SD: standard dosage; SR: standard regimen; LR: longer regimen; NDR: new drug regimen; IR: individualized regimen; N/A: not available; RCT: randomized controlled trial; RC: retrospective cohort study; PC: prospective cohort study; WHO: World Health Organization; IUATLD: International Union Against Tuberculosis and Lung Disease; NTP: National TB Control Program | | | | | | | | | | | | | | | | | | | | | | | | | | | | | | | | | | | | | | | | | | | | | | | | | | |
