# Supplementary material for: The efficacy and safety of high-dose isoniazid-containing therapy for multidrug-resistant tuberculosis: a systematic review and meta-analysis
Source: Front Pharmacol. 2024 Jan 8;14:1331371. doi: 10.3389/fphar.2023.1331371 (PMC10800833; doi:10.3389/fphar.2023.1331371)
Supplement: Supplementary file 1 [file DataSheet1.zip › Table S1.DOCX]

| Table S1. Baseline characteristics of eligible studies. | | | | | | | | |  |
| --- | --- | --- | --- | --- | --- | --- | --- | --- | --- |
| Study characteristics | |  | | Outcomes |  |  |  |  |  |
| No. | Author, year | Isoniazid dosage | Treatment regimen | Success (n) | Unsuccess (n) | Adverse events (n) | Culture conversion (n) | Conversion time (median, months) | Follow-up (n) |
| 1 | Katiyar, 2008 | Hh: 16-18 mg/kg  CG: 5 mg/kg or none | Hh: HhKLPCsPas (n-=42) CG: HKLPCsPas (n=40) or KLPCsPas (n=41) | N/A | N/A | N/A | Hh: 6m: 31/42 CG (nomall dose): 6m: 18/40 CG (placebo): 6m: 20/41 | Hh: 3.4 CG (nomall dose): 6.4 CG (placebo): 6.6 | N/A |
| 2 | Van Deun, 2010 | Hh: <33 kg, 300 mg 33-55 kg, 400 mg  >55 kg, 600 mg CG: <33 kg, 200 mg 33-55 kg, 300 mg  >55 kg, 300 mg | Hh: 4KCGEHhZP/5GEZC (n=206) CG: 3KCOEHZP/12OHEZC or 3KOEZP/12OEZP (n=221) | Hh: success: 181/206 cure: 170/206 completion: 11/206 CG: cure: 153/221 | Hh: unsuccess: 25/206 death: 11/206 default: 12/206 failure:1/206 relapse:1/206 CG: Unsuccess: 68/221 death: 22/221 default: 29/221 failure: 16/221 relapse: 0 other: 1/221 | Hh: 76/206 CG: 187/221 | N/A | N/A | 181 cure (24m): culture-negative: 165; LTFU: 7; death: 8; reinfection: 1 |
| 3 | Piubello, 2014 | <33 kg, 300 mg 33-55 kg, 400 mg  >55 kg, 600 mg | 4KCGEHhZP/5GEZC (n=65) | 58/65 | Unsuccess: 7/65 death: 6/65 default: 1/65 | 41/65 | 4m (culture): 61/62 6m (culture): 62/62 | N/A | 58 cure (24m): culture-negative: 49; LTFU: 4; death: 5; |
| 4 | Trébucq, 2018 | 10 mg/kg | 4KCMEHhZP/5MEZC (n=1006) | Success: 821/1006 cure: 728/1006 completion: 93/1006 | Unsuccess: 185/1006 death: 78/1006 failure: 59/1006 LTFU: 48/1006 | 897/1006 SAEs: 107/1006 | N/A | N/A | N/A |
| 5 | Harouna, 2019 | <33 kg, 300 mg 33-55 kg, 400 mg  >55 kg, 600 mg | 4KCGEHhZP/8GEZC (n=120) | 98/110 | 12/110 death: 9 failure:1 LTFU: 2 | 75/110 | N/A | N/A | 106 cure (12m): culture-negative: 88; relapse: 2 LTFU: 12; death: 4; |
|  |  |  |  | 8/10 | 2/10 death: 1 failure: 1 LTFU: 0 | 5/10 | N/A | N/A |  |
| 6 | Walsh, 2019 | Hh: 16–18 mg/kg  CG: none | Hh: 8KCMEHhZP/14KHh (n=99) CG: 8KMCsPasZP/12K or IR (n=88) | Hh: 88/99 CG: 70/88 | Hh: 11/99 CG: 18/88 | N/A | N/A | Hh: 1.75 CG: 2.28 | N/A |
| 7 | Zhdanova, 2021 | Hh: 600mg CG: none | Hh: 4K(or C)M(or Lfu)EHhZP/5KHh (n=132) CG: 8KMCsPasZP/12K (n=274)or IR (n=82) | Hh: success: 110/132 cure: 74/132 completion: 36/132 CG (SR): success: 137/274 cure: 89/274 completion: 48/274 CG (IR): success: 48/82 cure: 29/82 completion: 19/82 | Hh: unsuccess: 22/132 death: 0 failure: 4/132 LTFU: 18/132 CG (SR): unsuccess: 137/274 death: 36/274 failure: 8/274 LTFU: 93/274 CG (IR): unsuccess: 34/82 death: 4/82 failure: 12/82 LTFU: 18/82 | N/A | Hh: 2m: 62/132 4m: 79/132 6m: 81/132 CG (SR): 2m: 96/274 4m: 132/274 6m: 137/274 CG (IR): 2m: 38/82 4m: 44/82 6m: 50/82 | N/A | N/A |
| 8 | Pirmahmadzoda, 2021 | Hh: 10mg/kg CG: none | Hh: Cap(or A)CMEHhZP or Lzd(or Dlm)CMEHhZP (n=7) CG: Cap(or A)LCsPasZPFLD (n=39) or IR (n=12) | Hh: success: 7/7 cure: 2/7 completion: 5/7 CG (SR): success: 35/39 cure: 30/39 completion: 5/39 CG (IR): success: 12/12 cure: 8/12 completion: 4/12 | Hh: unsuccess: 0 CG (SR): unsuccess: 4/39 death: 2/39 failure: 1/39 LTFU: 1/39 CG (IR): unsuccess: 0 | N/A | N/A | N/A | N/A |
| 9 | Wahid, 2021 | 10-15mg/Kg | 4ACMEHhZEto/5MEZC (n=313) | Success: 262/313 cure: 250/313 completion: 12/313 | Unsuccess: 51/313 death: 31/313 failure: 4/313 LTFU: 16/313 | N/A | 2m: 221/313 | 1 | N/A |
| 10 | du Cros, 2021 | <25, 15-20 mg/kg;  25-32, 300mg;  33-50, 400mg;  >50, 600mg | 4KCMEHhZP/5MEZPC (n=128) | Success: 92/128 cure: 55/128 completion: 37/128 | Unsuccess: 36/128 death: 2/128 failure: 22/128 LTFU: 12/128 | 100/128 SAEs: 28/128 | N/A | N/A | 128 cure (12m): recurrence-free cure: 87; LTFU: 16; death: 2; failure: 22; recurrence: 1 |
| 11 | Trubnikov, 2021 | 10 mg/kg | 4CmCMEHhZP/5MEZPC (n=95) | 63/95 | Unsuccess: 32/95 death: 7/95 failure: 17/95 LTFU: 5/95 not evaluated: 3/95 | 38/95 SAEs: 21/95 | N/A | N/A | N/A |
| 12 | Mason , 2021 | N/A | 6KCMEHhZP/5MEZC (n=26) | 10/26 | Unsuccess: 16/26 death: 2/26 failure: 12/26 LTFU: 2/26 | 18/26 SAE: 10/26 | N/A | N/A | N/A |
| 13 | Koirala, 2021 | 10 mg/kg | 4K(or A)CMhEHhZP/5MEZPC (n=301) | Success: 239/301 cure: 177/301 completion: 62/301 | Unsuccess: 62/301 death: 36/301 failure: 16/301 LTFU: 8/301 not evaluated: 2/301 | 55/301 SAEs: 46/301 | 2m: 224/233 4m: 224/233 6m: 214/219 | N/A | N/A |
| 14 | Abubakar , 2022 | Hh: 10-15mg/Kg CG: none | Hh: IR with Hh (n=35) CG: IR without Hh (n=320) | Hh: 8/35 CG: 138/320 | Hh: 27/35 CG: 182/320 | N/A | Hh: 18m: 16/35 CG: 210/320 | N/A | N/A |
| 15 | Soeroto , 2022 | N/A | 4KCMEHhZEth/5MEZC (n=315) | 202/315 | Unsuccess: 113/315 death: 27/315 failure: 30/315 LTFU: 56/315 | N/A | 2m: 197/315 | N/A | N/A |
| 16 | Indarti , 2022 | N/A | Hh: 4KCMEHhZEto/5MEZC (n=65) CG: BdqCsZHPas (n=34) | Hh: success: 23/65 cure: 23/65 completion: 0 CG: success: 18/34 cure: 17/34 completion: 1/34 | Hh: unsuccess: 42/65 death: 6/65 failure: 2/65 LTFU: 32/65 not evaluated: 2/65 CG: unsuccess: 16/34 death: 4/34 failure: 1/34 LTFU: 10/34 not evaluated: 1/34 | N/A | N/A | N/A | N/A |
| 17 | Mleoh , 2023 | N/A | Hh: 4KCMEHhZP/5MEZC (n=160) CG: 8KCsLEZEto/12LEtoZCs±E (n=125) or 8K6Bdq/Dlm8LCsEtoLzdZ/12LEtoLzdCsZ (n=97) | Hh: success: 140/160 cure: 121/160 completion: 19/160 CG (LR): success: 90/125 cure: 79/125 completion: 11/125 CG (NDR): success: 74/97 cure: 68/97 completion: 6/97 | Hh: unsuccess: 20/160 death: 18/160 LTFU: 2/160 CG (LR): unsuccess: 35/125 death: 26/125 LTFU: 9/125 CG (NDR): unsuccess: 23/97 death: 18/97 LTFU: 5/97 | Hh: 53/160 CG (LR): 35/125 CG (NDR): 18/97 | N/A | N/A | N/A |
| 18 | Kumari, 2023 | N/A | 4KCMEHhZEto/5MEZC (n=360) | Success: 303/360 cure: 153/360 completion: 150/360 | Unsuccess: 37/360 death: 33/360 failure: 2/360 LTFU: 2/360 | 281/360 | N/A | N/A | N/A |
| 19 | Andrew J Nunn, 2019 | <33Kg, 300 mg 33-50Kg, 400 mg >50Kg, 600 mg | 4KCGEHhZP/5GEZC (n=253) | 193/245 | Unsuccess: 52/245 death:24 | SAEs: 136/282 | 2m (culture): 145/253 4m (culture): 247/253 6m (culture): 252/253 | N/A | N/A |
| Abbreviations: Hh: high-dose isoniazid group; CG: control group; SR: standard regimen; LR: longer regimen; NDR: new drug regimen; IR: individualized regimen; N/A: not available; SAEs: serious adverse events; H: isoniazid; A: amikacin; K: kanamycin; Cap: capreomycin; S: streptomycin; M: moxifloxacin; Mh: high-dose moxifloxacin; G: gatifloxacin; L: levofloxacin; O: ofloxacin; Cs: cycloserine; E: ethambutol; Z: pyrazinamide; P: prothionamide; Pas: para-aminosalicylic acid; C: clofazimine; R: rifampicin; Eto: ethionamide; Rpt: rifapentine; FQs: fluoroquinolones; Dlm: delamanid; Lzd: linezolid; Bdq: bedaquiline; FLD: first-line drugs; LTFU: Loss to follow-up | | | | | | | | | |
