# Supplementary material for: The efficacy and safety of high-dose isoniazid-containing therapy for multidrug-resistant tuberculosis: a systematic review and meta-analysis
Source: Front Pharmacol. 2024 Jan 8;14:1331371. doi: 10.3389/fphar.2023.1331371 (PMC10800833; doi:10.3389/fphar.2023.1331371)
Supplement: Supplementary file 1 [file DataSheet1.zip › Table S2.DOCX]

| Table S2. Quality assessment using Newcastle–Ottawa scale: observational studies. | | | | | | | | | | |
| --- | --- | --- | --- | --- | --- | --- | --- | --- | --- | --- |
| Study (cohort) | Selection | | | | Comparability | | Outcome | | | Quality score (9) |
|  | Representativeness of exposed cohort (1) | Selection of non-exposed cohort (1) | Ascertainment of exposure (1) | Demonstration that outcome of interest was not present at start of study  (1) | Comparability of cohorts on the basis of the design or analysis (1) | Comparability of cohorts on the basis of the measurement (1) | Assessment of outcome (1) | Was follow-up long enough for outcomes to occur  (1) | Adequacy of follow-up of cohorts  (1) |  |
| Van Deun, 2010 | 1 | 1 | 1 | 1 | 0 | 0 | 1 | 1 | 1 | 7 |
| Piubello, 2014 | 0 | 0 | 1 | 1 | 0 | 0 | 1 | 1 | 1 | 5 |
| Trébucq, 2018 | 1 | 0 | 1 | 1 | 1 | 0 | 1 | 1 | 0 | 6 |
| Harouna, 2019 | 0 | 0 | 1 | 1 | 0 | 0 | 1 | 1 | 1 | 5 |
| Walsh, 2019 | 0 | 1 | 1 | 1 | 1 | 0 | 1 | 1 | 1 | 7 |
| Zhdanova, 2021 | 1 | 1 | 1 | 1 | 0 | 0 | 1 | 1 | 1 | 7 |
| Pirmahmadzoda, 2021 | 0 | 1 | 1 | 1 | 0 | 0 | 1 | 1 | 1 | 6 |
| Wahid, 2021 | 1 | 0 | 1 | 1 | 1 | 0 | 1 | 1 | 1 | 7 |
| du Cros, 2021 | 0 | 0 | 1 | 1 | 1 | 0 | 1 | 1 | 1 | 6 |
| Trubnikov, 2021 | 0 | 0 | 1 | 1 | 1 | 0 | 1 | 1 | 1 | 6 |
| Mason , 2021 | 0 | 0 | 1 | 1 | 0 | 0 | 1 | 1 | 1 | 5 |
| Koirala, 2021 | 1 | 0 | 1 | 1 | 1 | 0 | 1 | 1 | 1 | 7 |
| Abubakar , 2022 | 1 | 1 | 1 | 1 | 1 | 0 | 1 | 1 | 1 | 8 |
| Soeroto , 2022 | 1 | 0 | 1 | 1 | 1 | 0 | 1 | 1 | 1 | 7 |
| Indarti , 2022 | 0 | 1 | 1 | 1 | 0 | 0 | 1 | 1 | 0 | 5 |
| Mleoh , 2023 | 1 | 1 | 1 | 1 | 1 | 0 | 1 | 1 | 1 | 8 |
| Kumari, 2023 | 1 | 0 | 1 | 1 | 0 | 0 | 1 | 1 | 1 | 6 |
| NOS: Newcastle-Ottawa Scale | | | | | | | | | | |
