# Supplementary material for: The efficacy and safety of high-dose isoniazid-containing therapy for multidrug-resistant tuberculosis: a systematic review and meta-analysis
Source: Front Pharmacol. 2024 Jan 8;14:1331371. doi: 10.3389/fphar.2023.1331371 (PMC10800833; doi:10.3389/fphar.2023.1331371)
Supplement: Supplementary file 1 [file DataSheet1.zip › Table S3.DOCX]

| Table S3. Quality assessment using Jadad scale: randomized controlled trials. | | | | | | | | | | |  |  |
| --- | --- | --- | --- | --- | --- | --- | --- | --- | --- | --- | --- | --- |
| Study (RCT) | Randomization | | | Concealment of allocation | | | Double blinding | | | Withdrawals and dropouts | | Quality score (7) |
|  | Not randomized or inappropriate method of randomization. (0) | The study was described as randomized.  (1) | The method of randomization was described and it was appropriate. (2) | Not describe the method of allocation concealment. (0) | The study was described as using allocation concealment method. (1) | The method of allocation concealment was described appropriately. (2) | No blind or inappropriate method of blinding. (0) | The study was described as double blind. (1) | The method of double blinding was described and it was appropriate. (2) | Not describe the follow-up. (0) | A description of withdrawals and dropouts. (1) |  |
| Katiyar, 2008 |  | 1 |  |  | 1 |  |  |  | 2 | 0 |  | 4 |
| Andrew, 2019 |  | 1 |  |  | 1 |  |  | 1 |  |  | 1 | 4 |
