# Supplementary material for: The efficacy and safety of high-dose isoniazid-containing therapy for multidrug-resistant tuberculosis: a systematic review and meta-analysis
Source: Front Pharmacol. 2024 Jan 8;14:1331371. doi: 10.3389/fphar.2023.1331371 (PMC10800833; doi:10.3389/fphar.2023.1331371)
Supplement: Supplementary file 1 [file DataSheet1.zip › Table S4.DOCX]

| Table S4. Pooled estimates for outcomes of all MDR-TB patients, stratified by high-dose INH and control groups. | | | | | | | | | | |
| --- | --- | --- | --- | --- | --- | --- | --- | --- | --- | --- |
| Treatment outcomes | Number of cohorts (n) | High-dose INH group | | | | Control group | | | | Meta-regression analysis |
|  |  | Events/total (n/N) | Proportions* (%; 95% CI) | I^2^ (%) | Egger's test | Events/total (n/N) | Proportions* (%; 95% CI) | I^2^ (%) | Egger's test |  |
| Treatment success | 10 | 814/1003 | 79.6 (67.7-89.5) | 93.5 | 0.34 | 775/1292 | 69.3 (58.7-79) | 92.8 | 0.04 | 0.22 |
| Cure | 8 | 587/869 | 60.4 (47.2-72.9) | 91.8 | 0.10 | 473/884 | 57.7 (43.4-71.4) | 93.6 | 0.50 | 0.50 |
| Completion | 7 | 120/663 | 20.3 (9-34.3) | 92.1 | 0.20 | 94/663 | 12.4 (7.1-18.9) | 74.9 | 0.91 | 0.49 |
| Treatment unsuccess | 10 | 189/1003 | 20.4 (10.5-32.3) | 93.5 | 0.34 | 517/1280 | 33.9 (24.1-44.4) | 92.7 | 0.08 | 0.18 |
| Death | 6 | 53/855 | 4.5 (0.8-10.5) | 91.0 | 0.93 | 110/833 | 12.8 (8.7-17.6) | 68.8 | 0.94 | 0.39 |
| Failure | 4 | 11/535 | 1.0 (0.2-1.9) | 48.0 | 0.03 | 37/611 | 6.3 (2.2-11.9) | 78.5 | 0.63 | 0.52 |
| Loss to follow-up | 5 | 72/649 | 11.7 (2-27.6) | 96.2 | <0.001 | 135/612 | 17.8 (6.4-32.9) | 94.0 | 0.47 | 0.59 |
| Adverse events | 3 | 182/526 | 34.6 (30.5-38.6) | 0.0 | - | 240/443 | 44.0 (6-87) | 98.9 | 0.12 | 0.65 |
| Culture conversion (2m) | 2 | 124/264 | 47 (40.9-53) | 0.0 | - | 134/356 | 40.6 (29.5-51.7) | 71.8 | - | 0.37 |
| Culture conversion (4m) | 2 | 158/264 | 60 (53.9-70.0) | 0.0 | - | 176/356 | 50.9 (40.0-56.9) | 0.0 | - | 0.33 |
| Culture conversion (6m) | 4 | 224/348 | 64.9 (59.9-69.8) | 38.0 | - | 225/437 | 53.5 (48.3-58.7) | 43.9 | 0.37 | 0.12 |
| *I²>50%, a random-effects model was employed, otherwise the fixed-effect model would be adopted. | | | | | | | | | | |
