# Supplementary material for: The efficacy and safety of high-dose isoniazid-containing therapy for multidrug-resistant tuberculosis: a systematic review and meta-analysis
Source: Front Pharmacol. 2024 Jan 8;14:1331371. doi: 10.3389/fphar.2023.1331371 (PMC10800833; doi:10.3389/fphar.2023.1331371)
Supplement: Supplementary file 1 [file DataSheet1.zip › Table S5.DOCX]

| Table S5. Subgroup analysis of sputum culture conversion at 6-month (with vs. without control group). | | | | | |
| --- | --- | --- | --- | --- | --- |
| Variables | Number of cohorts (n) | Events/total (n/N) | Culture conversion at 6-month (%, 95% CI) | I² (%) | P value |
| Control group |  |  |  |  |  |
| Yes | 2 | 112/174 | 64.5 (57.2-71.5) | - | 0.029* |
| No | 3 | 528/534 | 99.2 (97.6-100.0) | - |  |
| *Adj R-squared = 100.00% | | | | | |
