# Supplementary material for: The efficacy and safety of high-dose isoniazid-containing therapy for multidrug-resistant tuberculosis: a systematic review and meta-analysis
Source: Front Pharmacol. 2024 Jan 8;14:1331371. doi: 10.3389/fphar.2023.1331371 (PMC10800833; doi:10.3389/fphar.2023.1331371)
Supplement: Supplementary file 1 [file DataSheet1.zip › Table S6.DOCX]

| Table S6. Incidence of adverse events among high-dose INH group. | | | | | | | | | | | | | | | | |
| --- | --- | --- | --- | --- | --- | --- | --- | --- | --- | --- | --- | --- | --- | --- | --- | --- |
| Study | Cases (n) | Adverse events | Hepatotoxicity | Nephrotoxicity | Cardiotoxicity | Visual impairment | Ototoxicity | Arthralgia | Neurotoxicity | Electrolyte  disturbance | Psychiatric disorders | Dermatologic symptoms | Hematological disorders | Gastrointestinal symptom | Endocrine disorder | Others |
| Van Deun, 2010 | 206 | 76 | - | - | - | - | 13 | 2 | 8 | - | 1 | - | - | 44 | 8 | - |
| Piubello, 2014 | 65 | 41 | - | - | - | 2 | 13 | 4 | 3 | - | 3 | 2 | - | 22 | 6 | - |
| Trébucq, 2018 | 1006 | 897 | 491 | 158 | - | - | 446 | 183 | 271 | - | - | - | - | 574 | - | - |
| Harouna, 2019 (1) | 110 | 75 | 5 | - | - | - | 20 | - | - | - | - | - | - | 44 | - | - |
| Harouna, 2019 (2) | 10 | 5 | 1 | - | - | - | 1 | - | - | - | - | - | - | 3 | - | - |
| Walsh, 2019 | 99 | - | - | - | - | - | - | - | - | - | - | - | - | - | - | - |
| Zhdanova, 2021 | 132 | - | - | - | - | - | - | - | - | - | - | - | - | - | - | - |
| Pirmahmadzoda, 2021 | 7 | - | - | - | - | - | - | - | - | - | - | - | - | - | - | - |
| Wahid, 2021 | 313 | - | - | - | - | - | - | - | - | - | - | - | - | - | - | - |
| du Cros, 2021 | 128 | 100 | 2 | 7 | 2 | 1 | 5 | 7 | 9 | 1 | 2 | 6 | 2 | 45 | 1 | 10 |
| Trubnikov, 2021 | 95 | 38 | 8 | 4 | 4 | 1 | 7 | - | 2 | - | 1 | 7 | - | 12 | - | - |
| Mason , 2021 | 26 | 10 (SAEs) | 8 | - | 4 | - | 4 | - | - | 1 | - | - | - | - | - | 2 |
| Koirala, 2021 | 301 | 46 (SAEs) | 20 | 2 | 1 | 2 | 19 |  | 1 | 3 | 2 | 2 | - | - | 3 | - |
| Abubakar , 2022 | 35 | - | - | - | - | - | - | - | - | - | - | - | - | - | - | - |
| Soeroto , 2022 | 315 | - | - | - | - | - | - | - | - | - | - | - | - | - | - | - |
| Indarti , 2022 | 65 | - | - | - | - | - | - | - | - | - | - | - | - | - | - | - |
| Mleoh , 2023 | 160 | 53 | 2 | 5 | - | 2 | 13 | 2 | 9 | 1 | - | 2 | - | 17 | - | - |
| Kumari, 2023 | 360 | 281 | 9 | 25 | - | - | 17 | 26 | 29 | - | - | 10 | 6 | 159 | - | 38 |
| Andrew J Nunn, 2019 | 253 | 193 (SAEs) | 33 | - | 40 | - | 29 | - | - | 3 | - | - | - | - | - | - |
| Abbreviations: SAEs: serious adverse events; | | | | | | | | | | | | | | | | |
