# Supplementary material for: The efficacy and safety of high-dose isoniazid-containing therapy for multidrug-resistant tuberculosis: a systematic review and meta-analysis
Source: Front Pharmacol. 2024 Jan 8;14:1331371. doi: 10.3389/fphar.2023.1331371 (PMC10800833; doi:10.3389/fphar.2023.1331371)
Supplement: Supplementary file 1 [file DataSheet1.zip › Table S7.DOCX]

| Table S7. Subgroup analyses for adverse events among MDR-TB patients treated with anti-TB therapy containing high-dose INH. | | | | | | | |
| --- | --- | --- | --- | --- | --- | --- | --- |
| Factors | Number of cohorts | Events/total (n/N) | Adverse events (%) | 95% CI | I² (%) | Q test | P value |
| Study design |  |  |  |  |  |  |  |
| Prospective cohort study | 5 | 1395/1765 | 70.5 | 50-87.4 | 98.38 |  | 0.16 |
| Retrospective cohort study | 4 | 171/375 | 47.5 | 28.6-66.7 | 91.35 |  |  |
| Control group |  |  |  |  |  |  |  |
| Yes | 2 | 129/366 | 35.2 | 30.4-40.2 | - |  | 0.02* |
| No | 7 | 1437/1774 | 69.3 | 55.3-81.8 | 96.06 |  |  |
| *Adj R-squared = 58.69% | | | | | | | |
