# Supplementary material for: The efficacy and safety of high-dose isoniazid-containing therapy for multidrug-resistant tuberculosis: a systematic review and meta-analysis
Source: Front Pharmacol. 2024 Jan 8;14:1331371. doi: 10.3389/fphar.2023.1331371 (PMC10800833; doi:10.3389/fphar.2023.1331371)
Supplement: Supplementary file 1 [file DataSheet1.zip › Details for search strategies.DOC]

**1. Electronic search strategy**

All search run: June 25^th^, 2023

**2. Databases (searching syntax; records, n)**

**2.1 PubMed (n=493)**

((high AND dose* AND (INH OR isoniazid* OR isonicotin*)) OR "short course*" OR short-course OR "short regimen") AND (((MDR OR "multi drug resistan*" OR "multi-drug resistant*" OR multidrug-resistan*) AND (tubercu* OR TB)) OR MDR-TB) AND (treatment OR therapy OR outcome* OR efficacy OR effective* OR safe*)

**2.2 Embase (n=1339)**

((high AND dose* AND (INH OR isoniazid* OR isonicotin*)) OR 'short course*' OR short-course OR 'short regimen') AND (((MDR OR 'multi drug resistan*' OR 'multi-drug resistant*' OR multidrug-resistan*) AND (tubercu* OR TB)) OR MDR-TB) AND (treatment OR therapy OR outcome* OR efficacy OR effective* OR safe*)

**2.3 Scopus (n=1224)**

((high AND dose* AND (INH OR isoniazid* OR isonicotin*)) OR "short course*" OR short-course OR "short regimen") AND (((MDR OR "multi drug resistan*" OR "multi-drug resistant*" OR multidrug-resistan*) AND (tubercu* OR TB)) OR MDR-TB) AND (treatment OR therapy OR outcome* OR efficacy OR effective* OR safe*)

**2.4 Web of Science (n=513)**

((high AND dose* AND (INH OR isoniazid* OR isonicotin*)) OR "short course*" OR short-course OR "short regimen") AND (((MDR OR "multi drug resistan*" OR "multi-drug resistant*" OR multidrug-resistan*) AND (tubercu* OR TB)) OR MDR-TB) AND (treatment OR therapy OR outcome* OR efficacy OR effective* OR safe*)

**2.5 CINAHL (n=81)**

((high AND dose* AND (INH OR isoniazid* OR isonicotin*)) OR "short course*" OR short-course OR "short regimen") AND (((MDR OR "multi drug resistan*" OR "multi-drug resistant*" OR multidrug-resistan*) AND (tubercu* OR TB)) OR MDR-TB) AND (treatment OR therapy OR outcome* OR efficacy OR effective* OR safe*)

**2.6 Cochrane Library (n=60)**

((high AND dose* AND (INH OR isoniazid* OR isonicotin*)) OR ("short" NEXT course*) OR short-course OR "short regimen") AND (((MDR OR ("multi drug" NEXT resistan*) OR ("multi-drug" NEXT resistant*) OR multidrug-resistan*) AND (tubercu* OR TB)) OR MDR-TB) AND (treatment OR therapy OR outcome* OR efficacy OR effective* OR safe*)

**2.7 ClinicalTrials.gov (n=39)**

Intervention/Treatment: High-Dose Isoniazid
